# Physical Management of Scar Tissue: A Systematic Review and Meta-Analysis

**DOI:** 10.1089/acm.2020.0109

**Published:** 2020-10-08

**Authors:** Carlina Deflorin, Erich Hohenauer, Rahel Stoop, Ulrike van Daele, Ron Clijsen, Jan Taeymans

**Affiliations:** ^1^ Rehabilitation Research Laboratory (2rLab), Department of Business Economics, Health and Social Care, University of Applied Sciences and Arts of Southern Switzerland, Landquart, Switzerland.; ^2^ School of Sport, Health and Exercise Science, University of Portsmouth, Portsmouth, United Kingdom.; ^3^ International University of Applied Sciences THIM, Landquart, Switzerland.; ^4^ Faculty of Physical Education and Physiotherapy, Vrije Universiteit Brussel, Brussels, Belgium.; ^5^ Department of Rehabilitation Sciences and Physiotherapy, University of Antwerp, Antwerp, Belgium.; ^6^ Oscare, Organization for Burns, Scar After-Care and Research, Antwerp, Belgium.; ^7^ Department of Health, Bern University of Applied Sciences, Berne, Switzerland.

**Keywords:** cicatrix, conservative treatment, meta-analysis, physical therapy modalities, skin

## Abstract

***Objective:*** The aim of this systematic review with meta-analysis was to describe the status on the effects of physical scar treatments on pain, pigmentation, pliability, pruritus, scar thickening, and surface area.

***Design:*** Systematic review and meta-analysis.

***Subjects:*** Adults with any kind of scar tissue.

***Interventions:*** Physical scar management versus control or no scar management.

***Outcome measures:*** Pain, pigmentation, pliability, pruritus, surface area, scar thickness.

***Results:*** The overall results revealed that physical scar management is beneficial compared with the control treatment regarding the management of pain (*p* = 0.012), pruritus (*p* < 0.001), pigmentation (*p* = 0.010), pliability (*p* < 0.001), surface area (*p* < 0.001), and thickness (*p* = 0.022) of scar tissue in adults. The observed risk of bias was high for blinding of participants and personnel (47%) and low for other bias (100%).

***Conclusions:*** Physical scar management demonstrates moderate-to-strong effects on improvement of scar issues as related to signs and symptoms. These results show the importance of specific physical management of scar tissue.

## Introduction

Physical scar management represents an important field in science, as scars can negatively impact the quality of life of patients.^1,2^ Disturbing perceptions such as pain, tenderness or itchiness on the one hand, and functional limitations in the form of contractures on the other, are consequences of problematic scars. In addition, scar esthetics can also have a negative influence on psychosocial factors.^3–6^ The restoration of injured skin requires a complex sequence of physiological interactions to form appropriate scar tissue and repair the dermal lesion.^7^ Any dysfunction in the wound healing process may result in excessive scar tissue formation.^8^ Hypertrophic scars or keloids are the results of such deviant wound healing.^9^ Different therapy options, described in the literature include chemical, physical, and surgical methods.^10^ The physiotherapist focuses on conservative modalities in the treatment of scar tissue. These physical scar management options can be grouped into mechanotherapy, occlusive and hydrogenatic therapies, and light therapy, whereby often combinations are used.^11^ The purpose of physical scar management concentrates primarily on the prevention of an aberrant healing process of the skin.^12^ To date, the effects of physical scar management are still controversially discussed in literature and previous reviews focus on the treatment of hypertrophic scars and keloids after burn injuries.^11,13,14^ Consequently, the aim of this systematic review and meta-analysis was to evaluate the effectiveness of physical scar management on different symptoms in adults with any kind of scar tissue.

## Methods

### Research question

The research question was defined following the PICO model.^15^ Population: Adults with scar tissue; Intervention: Physical scar management; Comparator: Control intervention or no treatment; Outcome: Pain ratings, pigmentation, pliability, pruritus, scar surface area, and scar thickness. The choice for the outcome variables was based upon the fact that physical interventions will have a direct influence on gaining functional, physical, and psychological improvements^12^ and because these parameters show valuable signs that account for therapy progression.^16^

### Search strategy

An electronic search was conducted up to January 2020 on the databases PubMed (Medline), Cochrane Central Register of Controlled Trials (CENTRAL), and Physiotherapy Evidence Database (PEDro). Tissue-related keywords were combined with treatment-related keywords by using the Boolean operator “AND.” Tissue or treatment-related keywords themselves were combined with the function “OR.” The keywords used were proven for MeSH-terms (Table 1).

**Table 1.** Overview of Keywords and Combinations

Tissue related keywords		Treatment related keywords		Exclusion criteria
Scar (tissue) (MeSH)	AND	Casting physical activity	NOT	Surgery grafting
Burns (MeSH)		Compression (bandages)		
Cicatrix (MeSH)		Cream		
Keloid		Exercise		
Hypertrophic scar		Gel sheet(ing)		
		Hydration		
		Inserts		
		Laser therapy		
		Massage		
		Mechanical treatment		
		Mobilization		
		Moisturizer		
		Ointment		
		Physical treatment		
		Physiotherapy		
		Pressure therapy/garment		
		Rehabilitation stretching		
		Silicone gel		
		Skin cream		
		Splint(ing)		
		Tissue treatment		
		Topical treatment		
		Transdermal patch		

**FIG. 1.**
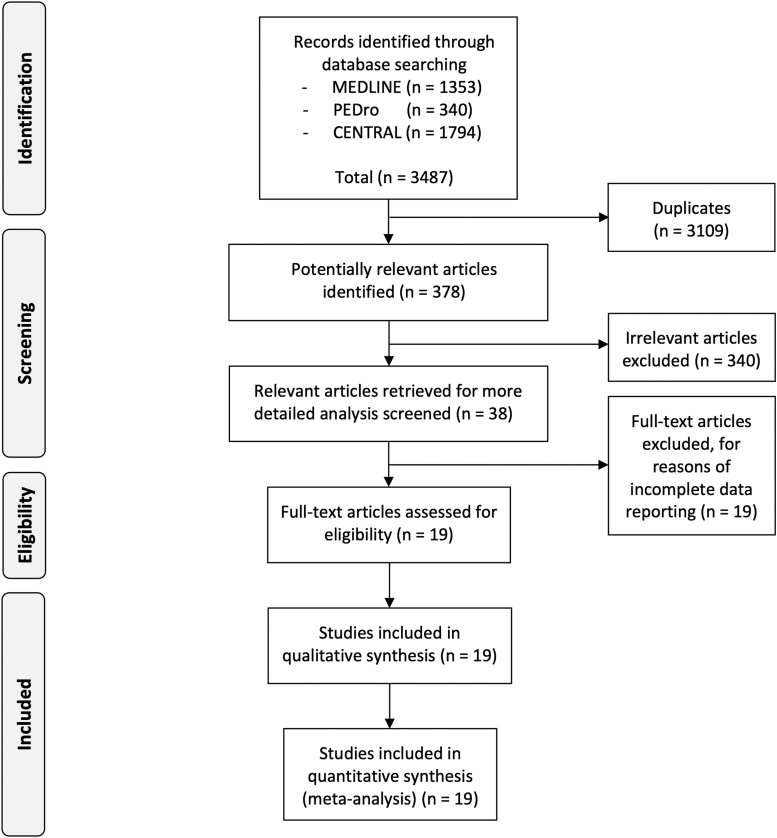
PRISMA flow chart describing the selection process.

The *a priori* set inclusion criteria were (1) randomized controlled trial, controlled clinical trial, or controlled trial, (2) German, French, Dutch, or English full-text availability, (3) human participants, (4) any kind of physical burn or scar tissue management, (5) control intervention or no treatment, (6) outcome parameters comprising pain and/or pruritus rating scales, subjective and/or objective scar/burn tissue evaluations. Title and abstracts of the (k = 3487) articles found were independently screened by three researchers (C.D., E.H., R.S.). A total of 19 articles were eligible for this meta-analysis (Fig. 1).

### Data extraction and quality assessment

Data were manually extracted from the included studies by three researchers (C.D., E.H., R.S.), independently from each other. In case of disagreement, a fourth researcher (R.C.) checked the variable and agreement was sought by consensus. The methodological quality of the studies was assessed with the Cochrane Risk of Bias Tool (Version 1).^17^ Two researchers independently evaluated the 19 studies (E.H., R.S.). A third researcher (R.C.) rated in case of disagreement.

### Data analysis

Comprehensive Meta-Analysis II software (CMA II; Biostat, Inc., Englewood, NJ) was used to conduct the meta-analysis calculations. A random-effects model was used to account for the fact that the included studies were not exact replicates of each other. Weighting factors were calculated based on the DerSimonian and Laird inversed-variance method.^18^ As most of the eligible studies used small samples, the individual studies' effect sizes were standardized and expressed as Hedges' g. The corresponding 95% confidence intervals (95% CI) around the individual studies' effect sizes as well as around the overall weighted estimate were calculated. Cohen's benchmarking for effect size interpretation was followed: g < 0.20 (negligible effect), g between 0.20 and 0.49 (small effect), g between 0.50 and 0.79 (moderate effect), and g > 0.80 (large effect).^19^ The null hypothesis of no heterogeneity (i.e., that all studies have a common effect size) was tested using the Cochran's Q-test. The Q-value, the corresponding degrees of freedom (df(Q)) as well as the corresponding exact *p*-value were reported. Because it has been described that the Q-test has a low statistical power, we set the significance level of the Q-test at 10%, as suggested.^20^ Higgins' I^2^ value was calculated to evaluate the amount of the total observed variance that can be explained by the true effect between studies' variance (rather than random sampling error). Higgins suggested that benchmarking values for the interpretation of heterogeneity be followed: I^2^ around 25% (low), I^2^ around 50% (moderate), and I^2^ around 75% or more (high).^20^

If a study showed an extreme effect size, a sensitivity analysis was conducted by excluding this study from the meta-analysis. An extreme effect size was defined, when the study's CI does not overlap with the CI of the pooled effect. The likelihood for publication bias was not tested because of less than 10 studies included in the different meta-analyses.^21^

## Results

### Risk of bias analysis

**FIG. 2.**
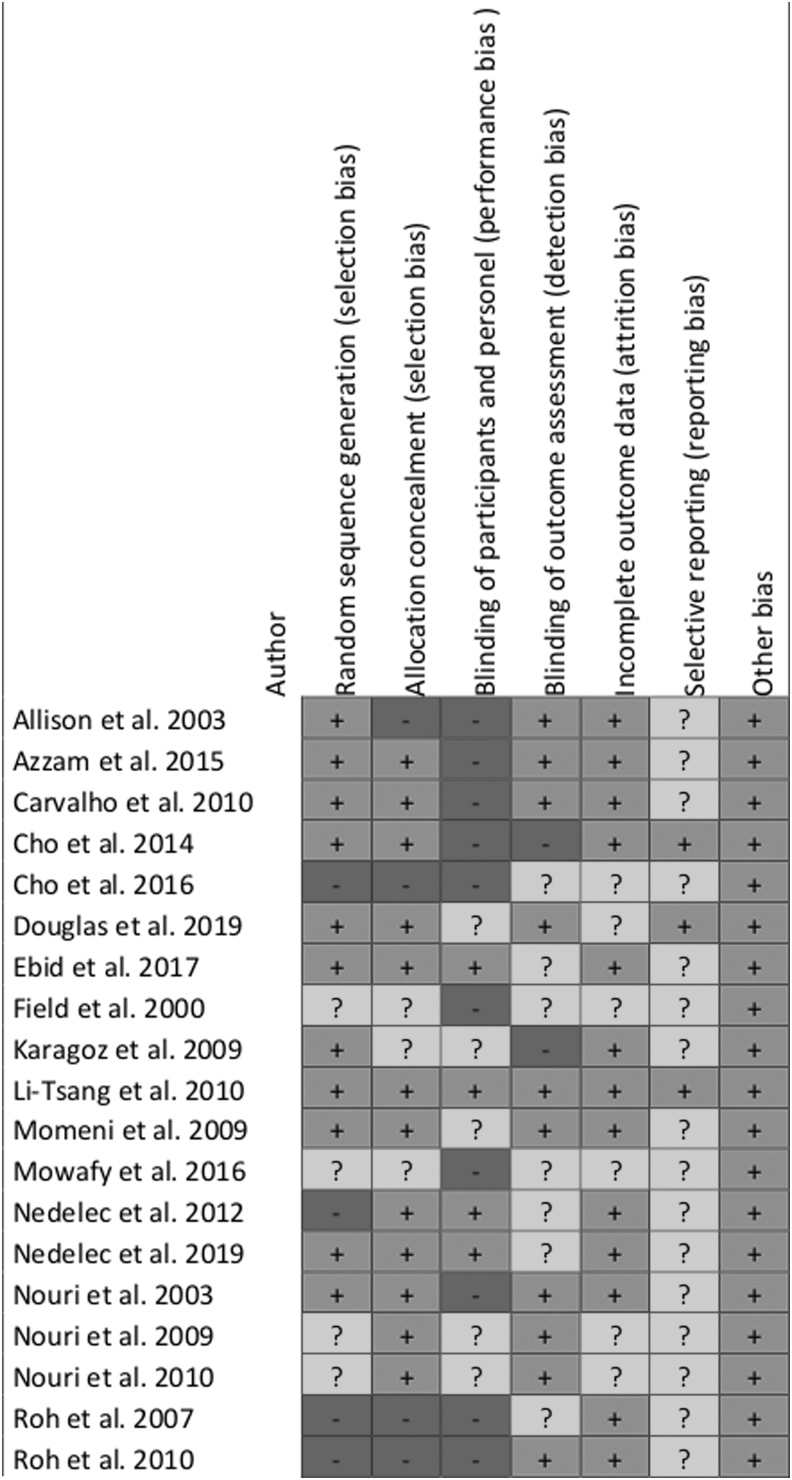
Risk of bias graph for each included study.

**FIG. 3.**
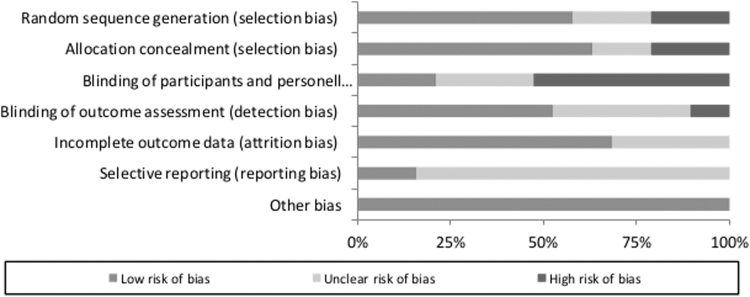
Risk of bias summary for all included studies.

The risk of bias analysis demonstrated a high risk for performance bias with 47% (blinding of participants and personnel). The reporting bias stayed unclear in 84% of the observed studies due to unclear or insufficient information. A low risk of selection (63%), attrition (68%), and other bias (100%) could be observed throughout the studies (Figs. 2 and 3).

### Study characteristics

In the present work, 13 studies investigated burn scars. These included seven studies with burn scars,^22–29^ five with hypertrophic burn scars,^28,30–33^ and one with hypertrophic scars after burns, scalds, or other skin traumata.^34^ Five studies focused on postsurgical scars,^35–39^ of which one concentrated specifically on hypertrophic and keloid scars.^39^ Finally, one study included all kinds of hypertrophic and keloid scars.^40^ Seven studies divided the scars into two or three halves to perform the intervention and control treatment on the same subject,^13,29,32,36–38,40^ whereas the other 12 studies analyzed scars of independent groups of subjects.^22,24–28,30,31,33–35,41^ The intervention methods can be categorized into (1) mechanotherapy (massage,^26–28,30,33^ extracorporeal shockwave therapy (ESWT)^24,25^), (2) occlusion and hydration therapy (silicone application,^31,32,34^ moisturizing cream with protease enzymes^41^), and (3) light therapy (noninvasive laser^22,29,35–40^). Table 2 gives a summary of the included studies.

**Table 2.** Characteristics of the Included Studies

Author year	Sample size,* n *Population specification	Control condition	Intervention versus control treatment	Outcome variable and assessment tool
Alster and Williams (1995)^39^	*n* = 16 (10 m, 6 f) FA IG *n* = 16, CG *n* = 16	Hypertrophic and keloidal postsurgical scars Scar divided into two halves	IG: PDL CG: no treatment	Pliability: Likert scale, Surface area: Magiscan digital image processing system, Thickness: caliper
Azzam et al. (2016)^40^	*n* = 30 (15 m, 15 f) FA IG *n* = 30, CG *n* = 30	Hypertrophic and keloidal scars Scar divided into two halves	IG: CO_2_ laser CG: no treatment	Surface area: biopsy
Carvalho et al. (2010)^35^	*n* = 30 FA IG *n* = 14, CG *n* = 15	Postsurgical scars Independent subject groups	IG: LLLT GA1As CG: no treatment	Pain: VAS
Cho et al. (2014)^30^	*n* = 146 FA IG *n* = 76 (61m, 15 f), CG *n* = 70 (50 m, 20 f)	Hypertrophic scars after burn Independent subject groups	IG: massage therapy and standard therapy CG: standard therapy alone	Pain: VAS Pruritus: itching scale Thickness: ultrasonography
Cho et al. (2016)^24^	*n* = 43 FA IG *n* = 20, CG *n* = 20 (34 m, 6 f)	Burn scars Independent subject groups	IG: ESWT and standard therapy CG: sham and standard therapy	Pain: NRS
Douglas et al. (2019)^29^	*n* = 19 (15 m, 4 f) FA IG *n* = 19, CG *n* = 19	Burn scars Scar divided into two halves	IG: CO_2_ laser CG: standard therapy	Pain: VSS Pruritus: VSS
Ebid et al. (2017)^22^	*n* = 49 (30 m, 19 f) FA IG *n* = 24, CG *n* = 25	Burn scars Independent subject groups	IG: pulsed HILT and standard therapy CG: placebo HILT and standard therapy	Pain: VAS
Field et al. (2000)^27^	*n* = 20 (14 m, 6 f) FA IG *n* = 10, CG *n* = 10	Burn scars Independent subject groups	IG: massage therapy and standard therapy CG: standard medical care	Pain: VAS
Karagoz et al. (2009)^31^	*n* = 32 (12 m, 20 f) FA IG 15 scars, IG 15 scars	Hypertrophic scars after burn Independent subject groups	IG: silicone gel CG: topical onion extract	Pigmentation: VSS Pliability: VSS Thickness: VSS
Li-Tsang et al. (2010)^34^	*n* = 104 (63 m, 41 f) FA IG *n* = 22, CG *n* = 12	Hypertrophic scars after burns, scalds, or other skin trauma Independent subject groups	IG: silicone gel sheet and lanolin massage CG: lanolin massage	Pain: VAS Pliability: VSS Pruritus: VAS Thickness: TUPS
Momeni et al. (2009)^32^	*n* = 38 (16 m, 18 f) FA IG *n* = 38, CG *n* = 38	Hypertrophic scars after burn Scar divided into two halves	IG: silicone gel sheet CG: placebo sheet	Pain: VSS Pliability: VSS Pruritus: VSS Pigmentation: VSS
Mowafy et al. (2016)^25^	*n* = 30 (m, f NM) FA IG *n* = 15, CG *n* = 15	Burn scars Independent subject groups	IG: ESWT and medical treatment CG: medical treatment alone	Pain: VAS
Nedelec et al. (2012)^41^	*n* = 23 FA IG *n* = 9, CG *n* = 9	Burn scars Independent subject groups	IG: moisturizer with Provase^®^ CG: standard moisturizer	Pruritus: VAS
Nedelec et al. (2019)^28^	*n* = 70 (41 m, 29 f) FA IG *n* = 70, CG *n* = 70	Hypertrophic scars after burn Independent subject group	IG: massage therapy CG: usual care	Pigmentation: light absorption Thickness: ultrasonography
Nouri et al. (2003)^37^	*n* = 11 (4 m, 7 f) FA IG *n* = 11, CG *n* = 11	Postsurgical scars Scar divided into two halves	IG: PDL and adhesive tape CG: no treatment and adhesive tape	Pigmentation: VSS Pliability: VSS Thickness: VSS
Nouri et al. (2009)^38^	*n* = 15 (11 m, 4 f) with 21 scars in total FA *n* = 14 with 19 scars	Postsurgical scars Scar divided into three parts	IG: PDL CG: no treatment	Pigmentation: VSS Thickness: VSS
Nouri et al. (2010)^36^	*n* = 20 (10 m, 4 f) FA IG *n* = NM, CG *n* = NM	Postsurgical scars Scar divided into three parts	IG: PDL CG: no treatment	Pliability: VSS Thickness: VSS
Roh et al. (2007)^33^	*n* = 35 (26 m, 9 f) FA IG *n* = 18, CG *n* = 17	Hypertrophic scars after burn Independent subject groups	IG: SRMT CG: no treatment	Pliability: VSS Thickness: VSS
Roh et al. (2010)^26^	*n* = 26 (24 m, 2 f) FA IG *n* = 13 (12 m, 1 f), CG *n* = 13 (12 m, 1 f)	Burn scars Independent subject groups	IG: SRMT CG: standard treatment	Thickness: ultrasonography

CG, control group, CO_2_, carbon dioxide, ESWT, extracorporeal shockwave therapy, f, females, FA, final analysis, HILT: high-intensity laser therapy, IG, intervention group, LLLT GA1As, low-level laser therapy, m, males, *n*, number of subjects, NRS, numeric rating scale, PDL: pulsed dye laser, SRMT, skin rehabilitation massage therapy, TUPS: tissue ultrasound palpation system, VAS, visual analog scale, VSS, Vancouver scar scale.

### Pain ratings

**FIG. 4.**
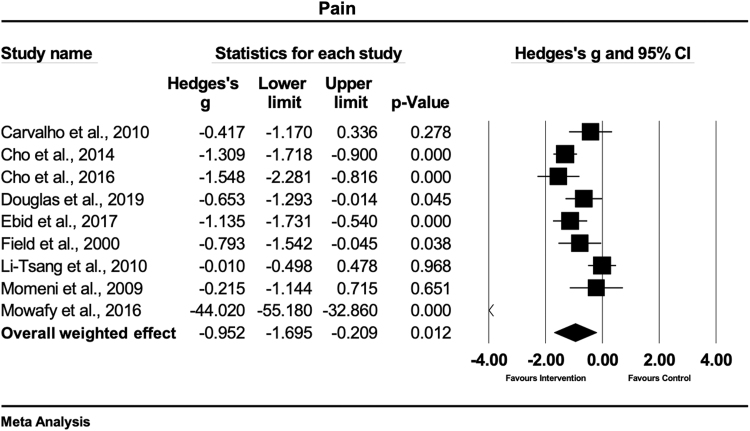
Forest plot of the meta-analysis illustrating the overall weighted effect size of physical therapy versus control on pain in patients with scar tissue. The *diamond* on the bottom of the forest plot represents the overall weighted estimate. CI, confidence interval.

Pain ratings were evaluated in nine studies.^22,24,25,27,29,30,32,34,35^ Of these, six studies measured pain with the visual analog scale (VAS),^22,25,27,30,34,35^ two applied the Vancouver scar scale (VSS),^29,32^ and one the numeric rating scale.^24^ Two studies each treated the scars with massage therapy,^27,30^ ESWT,^24,25^ laser therapy,^22,35^ and gel sheets,^32,34^ and one study with a CO_2_ laser.^29^ To test the hypothesis that physical therapy interventions had an enhancing effect to decrease pain as compared with control, an overall meta-analysis was conducted. Figure 4 shows that, in this set of sampled studies, physical scar management had a large and statistically significant effect on pain reduction compared with the control interventions (Hedges' g = −0.95 [95% CI: −1.69 to −0.20]). The observed heterogeneity was high and statistically significant (Q = 81.5; df(Q) = 8; *p* < 0.001; I^2^ = 90.1%) suggesting that about 90.1% of the variance of observed effects reflect the variance of true effects.

A sensitivity analysis was conducted since one study showed an extreme effect size.^25^ The results of the sensitivity analysis show a moderate and statistically significant effect on pain reduction compared with the control interventions (Hedges' g = −0.77 [95% CI: −1.18 to −0.36]). The sensitivity analysis decreased the observed heterogeneity, but remained high and statistically significant (Q = 23.95; df(Q) = 7; *p* = 0.001; I^2^ = 70.77%).

### Pigmentation

**FIG. 5.**
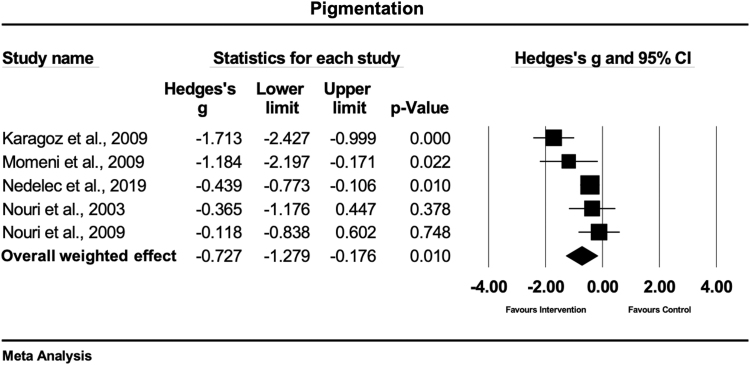
Forest plot of the meta-analysis illustrating the overall weighted effect size of physical therapy versus control on pigmentation in patients with scar tissue. The *diamond* on the bottom of the forest plot represents the overall weighted estimate.

Five studies investigated the effects of physiotherapy on pigmentation, using the VSS.^28,31,32,37,38^ One study treated scars with massage therapy,^28^ two with silicone applications,^31,32^ while two studies used laser therapy.^37,38^ Figure 5 shows that, based on this set of studies, there is evidence that physical scar management has a moderate and statistically significant effect compared with the control treatment on pigmentation (Hedges' g = −0.72 [95% CI: −1.27 to −0.17]). The heterogeneity was moderate and statistically significant (Q = 13.5; df(Q) = 4; *p* = 0.009; I^2^ = 70.4%).

### Pliability

**FIG. 6.**
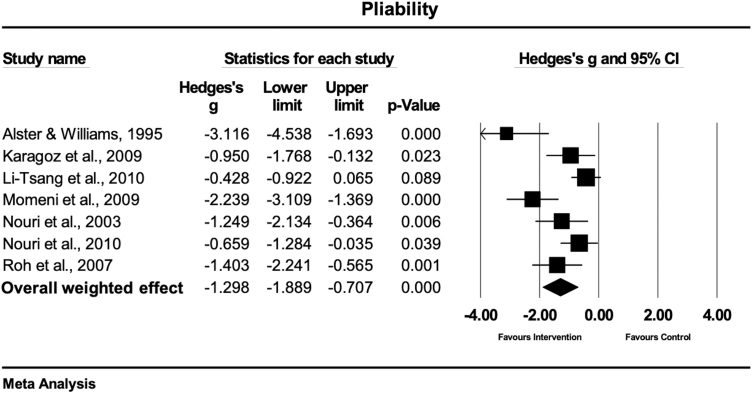
Forest plot of the meta-analysis illustrating the overall weighted effect size of physical therapy versus control on pliability in patients with scar tissue. The *diamond* on the bottom of the forest plot represents the overall weighted estimate.

Pliability was evaluated in seven studies.^31–34,36,37,39^ Six studies used the VSS,^31–34,36,37^ while one study used a 5-point Likert scale,^39^ for the quantification of pliability. Three studies treated the scars with laser therapy,^36,37,39^ three studies with gel sheets,^31,32,34^ and one study with massage therapy.^33^ The overall weighted effect size of physical (conservative) treatment as compared with control treatment in this set of scar studies, was large and statistically significant (Hedges' g = −1.29 [95% CI: −1.88 to −0.70]), favoring the physical scar management (Fig. 6). The observed heterogeneity was moderate but statistically significant (Q = 23.7; df(Q) = 6; *p* = 0.001; I^2^ = 74.7%).

### Pruritus

**FIG. 7.**
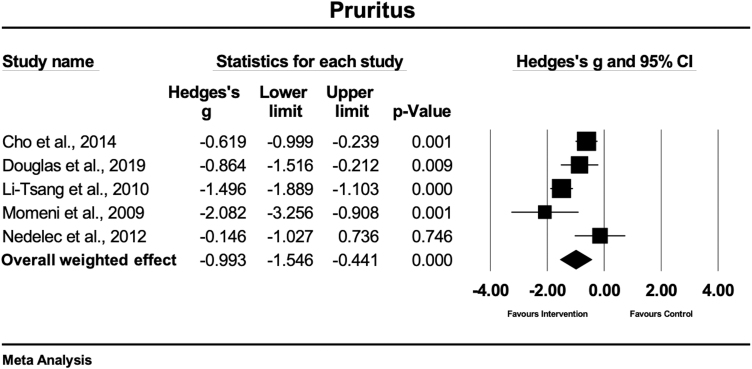
Forest plot of the meta-analysis illustrating the overall weighted effect size of physical therapy versus control on pruritus in patients with scar tissue. The *diamond* on the bottom of the forest plot represents the overall weighted estimate.

Five studies compared the effects between physical scar management and a control condition on pruritus.^29,30,32,34,41^ Two studies evaluated pruritus with the VSS,^29,32^ while the other studies used a VAS^34,41^ or an itching scale.^30^ Two studies treated the scars with gel sheets,^32,34^ one study each with massage therapy,^30^ CO_2_ laser therapy,^29^ and moisturizing cream with protease.^41^ In this sampled set of studies, the overall weighted effect revealed that conservative therapy had a large and statistically significant effect on pruritus compared with the control condition (Hedges' g = −0.99 [95% CI: −1.54 to −0.44]) (Fig. 7). The heterogeneity of the included studies was high and significant (Q = 17.0; df(Q) = 4; *p* = 0.002; I^2^ = 76.4%).

### Surface area

**FIG. 8.**
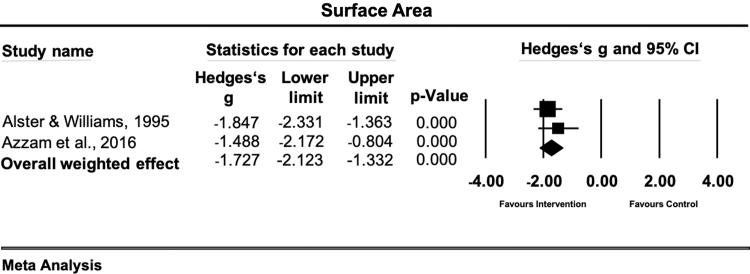
Forest plot of the meta-analysis illustrating the overall weighted effect size of physical therapy versus control on surface area in patients with scar tissue. The *diamond* on the bottom of the forest plot represents the overall weighted estimate.

Two studies met the inclusion criteria of evaluating the effects of conservative therapy versus a control condition on the surface area of the scar.^39,40^ All of them used different assessment methods comprising Magiscan digital image processing system^39^ and scar tissue biopsy.^40^ Each study applied laser therapy as physical scar management. The overall weighted effect size in this sample of studies was large and statistically significant (Hedges' g = −1.72 [95% CI: −2.12 to −1.33]) (Fig. 8). The observed heterogeneity was low and statistically not significant (Q = 0.70; df(Q) = 1; *p* < 0.401; I^2^ = 0.0%).

### Scar thickness

**FIG. 9.**
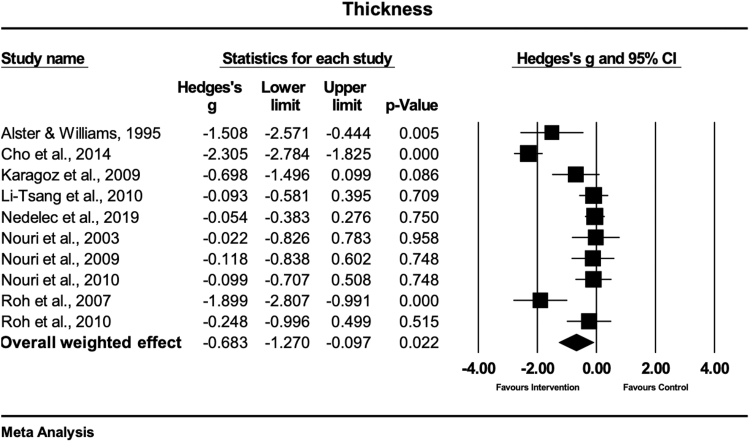
Forest plot of the meta-analysis illustrating the overall weighted effect size of physical therapy versus control on scar thickness in patients with scar tissue. The *diamond* on the bottom of the forest plot represents the overall weighted estimate.

A total of 10 studies evaluated the effects of physical scar management versus the control condition of scar thickness.^26,28,30,32,33,34,36–39^ The evaluation techniques were ultrasonography,^26,28,30^ tissue ultrasound palpation system,^34^ VSS subscale height,^31,33,36–38^ and caliper measurements.^13,39^ Four studies treated the scars with laser therapy,^36–39^ four studies with massage therapy,^26,28,30,33^ and two studies with gel sheets.^31,34^ The meta-analysis of the effect sizes extracted from this set of studies showed that physical scar management had a moderate and statistically significant effect on scar thickness reduction compared with the control group (Hedges' g = −0.68 [95% CI: −1.27 to −0.09)] (Fig. 9). The observed heterogeneity was high and statistically significant [Q = 81.0; df(Q) = 9; *p* < 0.001; I^2^ = 88.8%].

## Discussion

The aim of this review and meta-analysis was to evaluate the effectiveness of physical scar management on scar tissue. The main results of this analysis show significant overall effects in favor of the physical scar management compared with a control treatment for pain, pigmentation, pliability, pruritus, scar area, and thickness in adults suffering from any type of scar tissue.

However, this study has potential limitations. Although the performed literature search was conducted in three scientific databases, the authors are aware that publication bias might have occurred because no gray literature was screened. Insufficient data reporting might have led to potential over- or underestimation of the true effect as some authors did not show exact values (e.g., mean ± SD) but present the results as graphs only. Most of the included studies used small sample sizes, which limits the translation of these results into practice.

### Pain

The main results of this meta-analysis reveal that physical scar management as compared with a control treatment has a significant positive influence on pain ratings (Hedges' g = −0.95 [95% CI: −1.69 to −0.20]). The strongest effects were seen in two studies,^24,25^ both using a combination of ESWT and medical or standard treatment. Both ESWT treatments were conducted according to the guidelines of the international society of medical shockwave therapy, with ESWT of 100 impulses per cm^2^ on the affected location. These strong effects (Hedges' g range = −1.54 to −44.02) were seen in patients suffering from burn scars. The combination of ESWT and medical treatment^25^ demonstrated stronger effects compared with the combination of ESWT and standard therapy.^24^ The study from Mowafy et al.^25^ consisted of medication, physical therapy, and burn rehabilitation massage therapy while the content of the medical treatment of the other study from Cho et al.^24^ was not further described. A study showed an extreme effect size for ESWT.^25^ Therefore, we performed a sensitivity analysis where the study was excluded. The statistical significance of the overall effect of physical therapy management on pain reduction was maintained but changed from large to moderate (Hedges' g = −0.78 [95% CI: −1.19 to −0.37]).

A moderate-to-strong effect on pain reduction was observed in two studies, where scar tissue was treated with massage therapy for the duration of 30 min.^27,30^ Both studies used massage therapy in combination with standard therapy in hypertrophic burn scars^30^ or burn scars,^27^ respectively. A possible explanation for the stronger effect of massage therapy in the study of Cho et al.^30^ (Hedges' g = −1.30) could be the higher treatment frequency of three times versus two times a week, as used in the study of Field et al.^27^ (Hedges' g = −0.79). Another possibility is that a mechanotherapy, such as massage, might lead to better results in hypertrophic compared with nonhypertrophic burn scars.

The results of our study indicate that mechanical therapies, such as ESWT and massage therapy, seem to have a positive effect on pain reductions in burn scar. The non-nociceptive mechanical stimuli can reduce pain through the stimulation of nociceptive fibers, which are known to transmit sharp, acute, diffuse, and burning pain sensations.^42–44^ However, these interpretations should be handled with care as both studies using ESWT demonstrated a high risk of bias.^24,25^

Different types of light therapy also demonstrated to be effective interventions for pain reductions in the treatment of burn scars.^22,29^ Using a CO_2_ laser led to a significant and moderate pain reducing effect (Hedges' g = −0.65),^29^ whereas pulsed high-intensity laser therapy (HILT) showed a significant and large pain reducing effect (Hedges' g = −1.13).^22^ Published studies already reported that the higher intensity and the greater depth reached by HILT might be one reason for its effectiveness to relieve pain compared with low-level laser treatments.^45–47^ The reduction of pain levels by HILT is probably based on the inhibition of Aδ- and C-fiber transmission^48^ and by enhancing the production and release of endorphins.^22^ The results of this analysis might reveal that the general effects of HILT might be strong in the management of burn scars for pain reduction. However, future studies should evaluate this issue for making a valid statement.

Small effects and nonsignificant treatment differences for pain reduction (*p* > 0.05) were seen for the treatments with silicone gel sheets combined with massage^34^ or placebo sheets.^32^

### Pigmentation

The main results of this analysis reveal that the physical scar management compared with control has a significant positive effect on pigmentation (Hedges' g = −0.72 [95% CI: −1.27 to −0.17]). With respect to the other study results, the strongest effects were seen when applying a silicone gel twice a day on hypertrophic burn scars (Hedges'g = −1.71).^31^ A possible explanation might be that silicone applications are believed to positively influence the scar tissue through wound hydration,^49^ which is probably one reason that explains the great acceptance of this therapy since the early 1980s.^9^ Similarly to silicone gel applications, silicone gel sheets demonstrated to have a large and positive effect (Hedges'g = −1.18) on pigmentation of hypertrophic burn scars.^32^ Hydration and occlusion are known positive effects of silicone applications, while occlusion regulates epidermal cytokine and growth factor production in scar tissue.^11,50^ A 5-min massage treatment in burn patients showed a small and significant positive effect (Hedges'g = −0.43; *p* = 0.01) on pigmentation and melanin.^28^ This positive effect might result from an immediate increase in skin blood flow microcirculation.^51^ Longer lasting positive effects could be explained by the increased concentration of local transforming growth factor TGF-beta 1 and endothelin 1 that stimulates myofibroblast survival through protein kinase B activation.^52^

### Pliability

The main results of this analysis show a significant positive effect on scar pliability with physical scar management compared with the control treatment (Hedges' g = −1.29 [95% CI: −1.88 to −0.70]). The largest effects were observed in studies, where pulsed dye laser (PDL) therapy was used to treat postsurgical scars.^36,37,39^ All studies used a laser wavelength of 585 nm with a pulse duration of 450 μs. However, the study of Alster and Williams^39^ used higher fluences per pulse (mean 7.0 J/cm^2^) compared with 4.0 and 3.5 J/cm^2^, respectively.^36,37^ Interestingly, PDL led to stronger effects on hypertrophic and keloid postsurgical scars,^39^ compared with early postsurgical scar treatment.^36,37^ The use of an adhesive tape in combination with the PDL treatment demonstrated an additional positive effect of the tissue pliability in postsurgical scar treatment.^37^

Silicone gel and silicone gel sheets demonstrated to be effective in the treatment of pliability in hypertrophic burn scars (*p* = 0.02 and *p* < 0.001).^31,32^ Nevertheless, the study of Li-Tsang et al.,^34^ using silicone gel sheets did not show significant positive pliability effects (*p* = 0.08).^34^ These nonsignificant results might be explained by the inclusion of other traumatic skin lesions beside hypertrophic burn scars, which could have negatively impacted the treatment's effectiveness.

Only one study investigated the effects of skin rehabilitation massage therapy on the pliability of burn scars, showing a large and significant effect (Hedges'g = −1.40, *p* = 0.001).^33^ A described reason for the massage to increase the pliability of scars could be the mechanical disruption of the fibrotic tissue. The application of mechanical stimuli can lead to changes in the expression of extracellular matrix proteins and proteases, and therefore may change the structural and signaling milieu.^53,54^

### Pruritus

In general, the present analysis shows a significant positive effect of physical scar management compared with control treatments in the management of pruritus (Hedges' g = −0.99 [95% CI: −1.54 to −0.44]). The strongest effect in the reduction of pruritus or itching was seen for hypertrophic burn scars, treated with silicone gel sheets (Hedges'g = −1.49 and −2.08, respectively).^32,34^ Furthermore, the moisturizing effect of the silicone gel sheets on the stratum corneum layer also demonstrated to be effective to reduce pruritus in other traumatic skin lesions.^34^ A key factor seems to be the regulation of epidermal cytokines and growth factor production, which are evoked from occlusive therapy.^50^ Another effective method showing large and significant effects (Hedges'g = 0.86) in the treatment of pruritus was achieved with CO_2_ laser.^29^ Manstein et al.^55^ reported that the relief of itching can be explained by the undamaged columns of the skin between the microthermal treatment zones in CO_2_ laser treatment, resulting in rapid epithelialization.^55^

Further treatment that positively influenced pruritus in hypertrophic scar tissue was rehabilitation massage therapy, containing effleurage, friction, and petrissage techniques (*p* = 0.001).^30^ The observed moderate effect of massage therapy on pruritus (Hedges'g = −0.61) might be explained by the gate theory by Melzack and Wall^56^ or due to the release of beta endorphin levels.^57^ No positive effects were observed for the use of moisturizing creams (including proteases) in the reduction of pruritus in burn scar tissue (*p* > 0.05).^41^

### Surface area

Physical scar management showed a large and significant positive effect on the scar surface area (Hedges' g = −1.72 [95% CI: −2.12 to −1.33]). Only two studies investigated the effects of laser therapy on scar surface area in patients suffering from hypertrophic and keloid scars.^39,40^ A stronger effect was found for the PDL treatment (Hedges'g = 1.84),^39^ in comparison to fractional CO_2_ laser (Hedges'g = 1.48)^40^ in the management of hypertrophic and keloid scars. The study using PDL investigated postsurgical hypertrophic and keloid scars, while the fractional CO_2_ laser study included hypertrophic and keloid scars from different origins. The different laser therapy specifications and treated scar types make a general recommendation for a specific treatment setting difficult. However, these results demonstrate that laser light therapy is a promising treatment option for reducing scar surface area, probably due to increased tissue repair processes^58^ and enhanced anti-inflammatory actions.^59^

### Scar thickness

Physical scar management has a significantly positive effect on scar thickness management compared with control interventions (Hedges' g = −0.68 [95% CI: −1.27 to −0.09]).

Scar thickening was significantly (*p* = 0.005) reduced when PDL (wavelength 585 nm, pulse duration 450 μs, and mean fluence per pulse of 7.0 J/cm^2^) was used in the treatment of hypertrophic and keloidal postsurgical scars compared with no treatment.^39^ However, these results do not corroborate the findings from other studies, using PDL to decrease scar thickening of the skin after postsurgical scars compared with no treatment.^36–38^ A reason for the different results might be due to the different laser settings. While one study used a fluence per pulse between 6.5 and 7.25 J/cm^2^,^39^ the other authors^36–38^ used lower pulses between 3.50 and 4.00 J/cm^2^. While in the study of Alster and Williams,^39^ the treated scar area was smaller compared with others (range: 7 to 10 mm),^36–38^ the totally applied laser energy applied was higher, which might have contributed to the significant positive effects.

Two studies demonstrated that massage therapy was effective in reducing scar thickening in hypertrophic and burn scars.^30,33^ However, also controversial results were found in our analysis. Two included studies, using skin rehabilitation massage in the treatment of hypertrophic and burn scars, showed no effects to reduce scar thickening.^26,28^ Besides the interindividual treatment intensities of massage therapy, the treatment frequency of the intervention might have also contributed to the different effects of massage therapy. While the two studies^30,33^ showing a positive effect of massage therapy on scar thickening used a treatment frequency of one to three times per week with a treatment duration of 30 min, the noneffective studies showed only treatment durations of 5 min^28^ or did not report^26^ the exact frequencies and durations. It seems that the combination of massage therapy with standard therapy (including joint mobilizations, silicone gel applications, *etc.*)^30^ has an additional beneficial effect compared with massage therapy alone^33^ (Hedges'g = −2.30 vs. −1.89).

## Conclusion

In general, this meta-analysis shows that physical scar management has a significant positive effect to influence pain, pigmentation, pliability, pruritus, surface area, and scar thickness compared with control or no treatment.

Treatment modalities, such as ESWT, massage, as well as high-intensity light therapy, seem to be most effective agents in reducing pain in burn scars. Regarding the treatment of pliability and scar thickness, positive effects were seen for the use of PDL in postsurgical scars, while silicone gel and silicone gel sheets demonstrated to be effective in the management of pliability in hypertrophic scars. Our study further revealed strong effects in the reduction of pruritus, using silicone gel sheets in hypertrophic burn scars. Scar thickness was positively affected when hypertrophic and burn scars were treated with massage therapy, while scar surface area was positively influenced by laser therapy modalities (PDL and CO_2_ laser).

To investigate the most effective physical therapy strategy, further studies are needed, evaluating head-to-head comparisons of different physical scar therapy modalities.
